# HSBM-Produced Zinc Oxide Nanoparticles: Physical Properties and Evaluation of Their Antimicrobial Activity against Human Pathogens

**DOI:** 10.1155/2022/9989282

**Published:** 2022-12-23

**Authors:** Imen Massoudi, Ridha Hamdi, Ibtisam Ababutain, Ethar Alhussain, Aya Kharma

**Affiliations:** ^1^Department of Physics, College of Science, Imam Abdulrahman Bin Faisal University, P.O. Box 1982, 31441 Dammam, Saudi Arabia; ^2^Basic & Applied Scientific Research Center, Imam Abdulrahman Bin Faisal University, P.O. Box 1982, 31441 Dammam, Saudi Arabia; ^3^Department of Biology, College of Science, Imam Abdulrahman Bin Faisal University, P.O. Box 1982, 31441 Dammam, Saudi Arabia

## Abstract

This work examines the antibacterial and anticandidal activities of zinc oxide nanoparticles (ZNPs) synthesized by high-speed ball milling (HSBM), for short milling times: 0.5, 1, 1.5, and 2 h. First, ZNPs have been characterized by X-ray diffraction (XRD), transmission electron microscopy (TEM), Fourier transform infrared spectroscopy (FT-IR), Raman spectroscopy, and the Zetasizer analyzer. The HSBM results in semispherical ZNPs with some local agglomeration. We found that nanoparticles decrease in size continuously with milling time until they reach about 84% of their original size after only two hours; at 1000 rpm, HSBM reduces ZNP's average size by 6 nm/min. As particle size decreases, the X-ray diffracted patterns become broader and less intense while confirming that no phase transformation has occurred, proving HSBM's effectiveness in synthesizing nanoparticles on a large scale within a short period of time. According to FT-IR analysis, as material sizes change, the polarization charge of the ZNP surface changes as well, creating discrepancies in vibrational frequency, as demonstrated by the shifting of the IR spectra in the 300–600 cm^−1^ frequency band. Raman responses have also been proven to depend on the particle size. Using the Agar well diffusion method, eleven microorganisms have been tested for the antimicrobial activity of ZNPs. Among the six Gram-negative tested bacteria, *S. sonnei* showed the largest inhibition zone of about 11.3 ± 0.6 mm with ZNPs measuring 148 nm in size (milled for 2 h), followed by E. *coli ATCC* 25922. Accordingly, *S. aureus* was the most susceptible Gram-positive bacteria, with inhibition zone size gradually increasing from 11.8 ± 0.3 mm to 13.5 ± 0.5 mm with decreasing nanoparticle size from 767 to 148 nm, while *S. aureus* ATCC 25923 was resistant to both milled and unmilled samples. Similar results were seen with candida, all milled ZNPs inhibited *C. albicans*, followed by *C. tropicalis*, whereas *C. knisei* was resistant to all ZNP sizes. In light of microorganism-ZNP interaction mechanisms, the obtained results have been discussed in depth.

## 1. Introduction

During the evolution of life on Earth, bacteria and fungi have evolved into highly adaptable species. Without a doubt, antibiotics and antifungals were one of the key findings of the 20^th^ century for mankind. Resistance to antibiotics and antifungal treatments, however, is a major contributing factor to the lack of effective antimicrobial agents. Several mechanisms can cause antimicrobial resistance, including the use of lactamases, aminoglycoside modifying enzymes, and membrane permeability. The overuse and/or misuse can also lead to antimicrobial resistance [1]. Additionally, these microorganisms can develop resistance through the combination of genetic mutations or by exchanging DNA or gene fragments with viruses [2]. The current era is characterized by new resistant human pathogenic microorganisms that cause prolonged infection periods and higher mortality rates. In light of this global challenge, the development of novel treatments is becoming more challenging even for the pharmaceutical industry. Thus, there is an urgent need for new substances with antimicrobial properties [3]. A recent report from the WHO suggests that the most dangerous microorganisms are multidrug-resistant, mainly Acinetobacter, Pseudomonas, and various Enterobacteriaceae including *Klebsiella*, *E. coli*, *Serratia*, and *Proteus* [4]. They can cause severe and often deadly infections such as bloodstream infections and pneumonia [5]. As it called upon the scientific research and development sectors to support the pharmaceutical industry efforts in developing effective therapeutic agents.

Nanoparticles (NPs) are a revolutionary type of antimicrobial agent with high targeting capabilities. Due to their ultrasmall size, large surface area to mass ratio, and enhanced chemical and physical reaction activity, they do not bind to specific receptors within the cells, as they inhibit the development of fungi/bacteria resistant strains by targeting multiple biomolecular mechanisms simultaneously. Hence, NPs work differently from traditional antibiotics and antifungals in terms of their mechanisms of action. Studies examining the efficacy of metallic nanoparticles (MNPs) on several microorganisms, both in vivo and in vitro, have been overwhelmingly positive thus far. Indeed, MNPs have enzyme-like activity due to their strong catalytic action enabling them to be applied in biosensing and food safety applications, as well as in environmental monitoring. For example, it has been demonstrated that gold nanoparticles can be used as carriers due to their low toxicity and strong absorption spectrum. They may be also useful for treating infections caused by a variety of bacteria, including antibiotic-resistant bacteria in the skin and the respiratory tract. Aside from this, gold nanoparticles have also been proposed as potential photothermal converters, calorimetric detectors, and catalytic and imaging materials. Lately, researchers have used silver and calcium phosphate nanoparticles in dental composites, adhesives, and coatings, as they exhibit antibiofilm, protein rejection, and anticapillary functions. In vitro experiments have shown that copper nanoparticles have antibacterial, antifungal, and antiviral properties. Not only that, but copper nanoparticles are also useful for dental materials, blood vessel transplants, treating burns and infections in hospitals, and preventing microbial growth. In addition to being widely used in drug delivery and food packaging, copper nanoparticles are also used in water filtration and treatment [6].

Studies have also been conducted to determine the ability of metal oxide nanoparticles to selectively overcome microbial species, such as TiO_2_, CuO, ZnO, MgO, BiVO_4_, Ag_2_O, and FeO [7]. As well as being used in electronic devices such as gas sensors and photoconductivity, these materials can also be applied in the biomedical field. Indeed, it has been demonstrated that microbial screening protocols with metal oxide NPs lead to the production of reactive oxygen species (ROSs) such as H_2_O_2_, which results in oxidative stress in the microorganisms [8]. For example, copper oxide NPs are useful in preventing inflammation, fabricating antimicrobial films for food packaging, and preventing radiation damage to cells and even can effectively treat secondary dental caries. Indeed, it has been reported that adding copper oxide NPs to dental adhesive can improve its shear bond strength and provide antimicrobial properties without increasing its inherent cytotoxicity. A growing number of studies have shown that cerium oxide nanoparticles exhibit antioxidant, antitumor, antibacterial, and angiogenesis properties. Similarly, magnesium oxide nanoparticle-enhanced binding membranes exhibit considerable antimicrobial and osteogenic properties. In this way, periodontal tissue regeneration is effectively guided. Even with this knowledge, it is still challenging to determine how metal oxide NPs can cause microorganism toxicity [9]. This is because the mechanism depends on the different physical properties and features of nanomaterials. This is of particular importance when tuning the NPs' size and surface crystallinity since this causes a significant change in their physicochemical activities [10].

Nanoparticles of zinc oxide (ZNPs) have recently been recognized as a promising option for many industries. Its energy gap at room temperature is 3.37 eV, making it a wide band-gap semiconductor. Physically, this material is very interesting due to its thermal stability, high electron mobility, piezoelectric properties, interesting transparency in the visible range, and excellent photocatalytic performance. As a result of their anticorrosive and antifungal properties, ZNPs are also able to be used as a UV filter. Moreover, ZNPs have been widely applied in a variety of areas, such as pharmaceuticals, automobiles, textiles, solar cells, and cosmetics. In terms of effectiveness and safety as a chemical entering the body circulation, zinc oxide is among the most effective metal oxides that exhibit strong antibacterial [11–13] and anticandidal properties [14, 15]. Furthermore, it is listed by the U.S. Food and Drug Administration (21CFR182.899) as a safe material [16]. Based on recent studies, ZNPs are reliable nanoparticles for controlling human pathogens, particularly oral pathogens.

To reduce the size of ZnO at the nanoscale level, a variety miscellany of synthetic top-down techniques is used.  Table 1 shows some examples of relevant techniques for ZNP fabrication and the corresponding tested microorganisms. In general, top-down techniques can be categorized into mechanical, chemical, or biological approaches. A variety of chemical processes have been used to fabricate nanomaterials with advanced physical properties, such as chemical vapor synthesis, coprecipitation, vacuum evaporation, hydrothermal synthesis, and microwave synthesis. Despite the advantage of these processes in terms of producing nanomaterials in less time, there are growing concerns over their adverse effects on the environment and public health. This is mainly due to the use of toxic chemicals. As well as being quite expensive, these methods are potentially dangerous as well. It is, therefore, crucial to develop environmentally friendly ways to produce ZNPs. In this way, the use of plant extracts and microorganisms as natural sources for zinc nanoparticle synthesis has been proposed as a promising alternative to chemical synthesis. However, most of these biological approaches require a long time to complete and thus are not suitable for large-scale production of ZNPs. Further, their complexity and reliance on low-energy sources make them susceptible to small changes in the environment, such as weather conditions, solar activity, and even cosmic ray exposure [17].

In contrast, physical and mechanical methods such as electrical arc discharge and ball milling have demonstrated remarkable results in nanoparticle production. In addition to being simple and efficient, these techniques are also able to produce large quantities of various brand new metastable materials that have not been possible to be synthesized via other methods. In the arc discharge technique, the medium is a key factor in controlling the chemical compositions, particle sizes, and crystal structures of fabricated NPs. Furthermore, NP yields are significantly affected by the current. In liquid media, increasing the current rate from 50 to 80 A/cm^2^ results in decreased NP sizes. In contrast, they appear to increase in size in gaseous media [18]. Comparatively, milling time is a key parameter in the ball milling process when it comes to phase formation, microstructural distribution, and homogeneous distribution of chemical components. Additionally, it affects the physicochemical-mechanical properties of composites. Furthermore, mechanical milling can be carried out under ambient conditions and with different types of mills. These include centrifugal, vibrational, attritor, and high- and low-speed tumbling mills [19–23]. A longer milling time is generally required to activate and complete the structural and chemical changes needed to produce the desired results. For this reason, there has always been a trend to mill ZnO nanoparticles for very long periods to be effective against some pathogenic bacteria and *Candida*, for example 24 hours in Reference [24] and 50 hours in Reference [25]. In contrast to conventional ball milling, high-speed ball milling (HSBM) results in a rapid phase reaction between solid reactants and size reduction due to the high speed of rotating balls placed under a high magnetic pulling force. As an example, HSBM can not only produce ultrafine nanoparticles with definite stoichiometric ratios, but also enable products with higher yields, enhanced densification, and homogeneity. As a result, industrial-scale manufacturing can be achieved quickly and easily with HSBM. However, through this route, during this process, segregation phenomena, such as the presence of reagent residues, are unavoidable. Due to this, synthesized NPs must undergo a dispersion process before being tested for antimicrobial activity.

In this paper, we use HSBM during short milling times (0.5, 1, 1.5, and 2 h) to synthesize ZnO nanoparticles. The yielded ZNPs are then characterized physically using a variety of methods, including X-ray diffraction (XRD), transmission electron microscopy (TEM), Fourier transform infrared spectroscopy (FT-IR), Raman spectroscopy, and Zetasizer analyzer instruments. The antimicrobial activity of products is then tested against different human pathogenic microorganisms including Gram-negative/positive bacteria and unicellular fungi. Based on a series of in vitro tests, we then discuss the antimicrobial effectiveness of ZNPs obtained by HSBM during short milling times. This research will increase the potential for HSBM applications in the biomedical industry.

## 2. Materials and Methods

### 2.1. Synthesis of Nanoparticles

To synthesize ZNPs, we used commercially available ZnO powders with a purity >99.5% (Sigma Aldrich). Planetary magnetic ball mills (Retsch's Emax) were employed for the milling. Rotation speeds were set at 1000 rpm for the disk and vial. The abrasion of the sample is prevented by the use of three high-quality zirconium oxide (ZrO_2_) grinding balls. We choose four different short milling times to prepare the samples: 0.5 h, 1 h, 1.5 h, and 2 h.

### 2.2. Preparation of ZNPs for Antimicrobial Activity Screening

**Figure 1:**
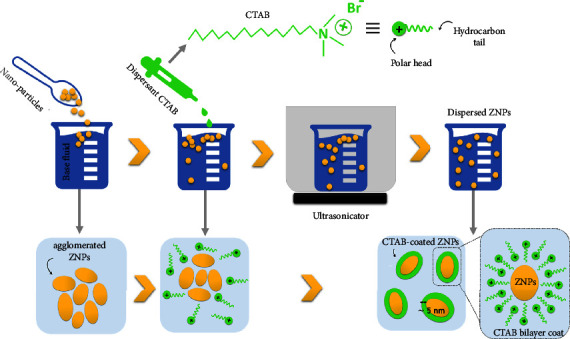
The conventional method for preparing ZNPs using CTAB dispersant.

It has been shown that MNPs are more toxic to microbes when dispersed in a base fluid [9]. The HSBM synthesis process is therefore followed by a dispersion process (Figure 1). A significant parameter in this process is the volume fraction of the NPs, as it influences the physicochemical behavior of the base fluid. For medical applications, nanoparticle fractions should range between 0.5 and 5% [26]. In our case, the most effective results have been obtained using zinc oxide volume fractions of less than 1% by weight. After that, an extremely low concentration of hexadecyl trimethyl ammonium bromide (CTAB), 0.4 *μ*M, is added as a surfactant to stabilize the ZNPs in the deionized water (base fluid). In general, CTAB is not toxic to human cells at concentrations less than 1 *μ*M. Next, to ensure the dispersion of ZnO, we sonicate ZNPs at room temperature for two hours with POWER SONIC ultrasound (US) 405. As illustrated in Figure 1, in this phase, reverse micelles are formed around the nanoparticle: the 16-carbon tails of the CTAB overlap to form a hydrophobic shell around the ZNP [26]. The reaction sequence is continued by the exchange of materials between reverse micelles and ZnO. In this way, ZNPs are dispersed and stabilized.

### 2.3. ZNPs Characterization

Nanoparticles undergo physical changes after the milling process. Therefore, using the FEI Titan G2 TEM (FEI, Morgagni 268) operating at 80 kV, we investigated their elemental composition. FT-IR measurements were conducted on a Perkin Elmer GX spectrometer equipped with a DTGS detector with a resolution of 2 cm^−1^. A Bruker FT-Raman spectrometer multi-RAM (Bruker Optics Inc., Billerica, MA, USA) with FT-Raman microscope II was used to conduct Raman measurements at room temperature, at an excitation of 532 nm. To determine the phase composition and crystallographic features of the powders before and after milling, we employ a Bruker AXS D8 advance X-ray diffractometer with Cu Ka (*λ* = 1.5418 Å) operating at 40 kV/40 mA and collecting from 2*θ* = 10 to 80°. Finally, we employ the Malvern Instruments Zetasizer analyzer (ZEN 3600) to measure the average size, electrophoretic mobility, and zeta potential of ZNPs.

### 2.4. Screening for Antimicrobial Activity

The five different samples of the studied ZNPs were screened for their antimicrobial activity at a concentration of 1 mg/ml using the Agar well diffusion method [27]. These samples were tested against eleven microorganisms kindly provided by King Fahd Hospital, Al Khobar, the kingdom of Saudi Arabia: *Candida knisei, Candida tropicalis, Candida albicans, Escherichia coli* ATCC25922*, Escherichia coli, Klebsiella pneumonia, Pseudomonas aeruginosa, Shigella sonnei, Staphylococcus aureus ATCC* 25923*, Staphylococcus aureus*, and *Streptococcus anginosus*. Inoculums of the tested microorganisms were prepared from 24 h cultures in Mueller Hinton broth. The turbidity of the inoculums was set to 1.8–0.63 × 10^8^ CFU/mL that representing 0.5–0.63 McFarland standards, using Biomerieux DensiCHEK Plus meter device. The preadjusted microorganism suspensions were then transferred, individually, to sterile Petri plates using the pour plate method. The Mueller Hinton Agar media was poured over the inoculums, and the plates were rotated to ensure even distribution, and then allowed to harden for 5–10 minutes. Seven wells were drilled using a sterile cork borer size of 8 mm. Each well was filled with 100 *μ*L of the tested ZNPs; as a negative control, the base fluid without nanoparticles was used, and as positive controls erythromycin (E15 mcg) and nystatin (100 mg) were used for bacteria and candida, respectively. The plates were then incubated at 37°C for 24 hours. The antimicrobial activity of the ZNPs was recorded in millimeters (mm) by measuring the areas with no microbial growth around the wells that appear as a clear inhibition zone. Three replicates of each experiment were carried out.

## 3. Results and Discussion

### 3.1. Characterization

#### 3.1.1. Microstructural Analysis by TEM

**Figure 2:**
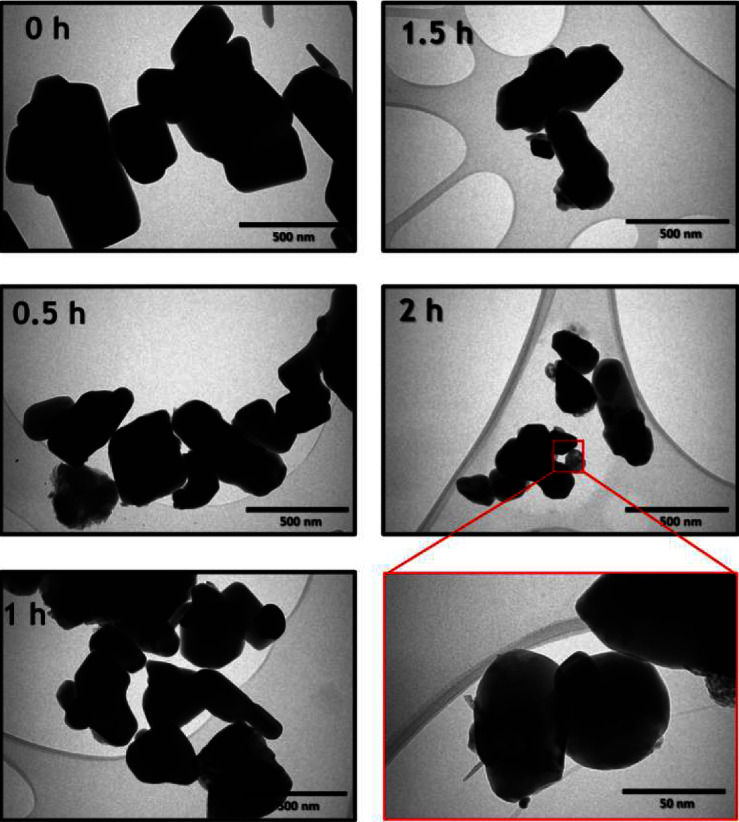
TEM micrographs of ZnO at different ball-milling times. 0 h refers to the unmilled ZnO powder.

**Figure 3:**
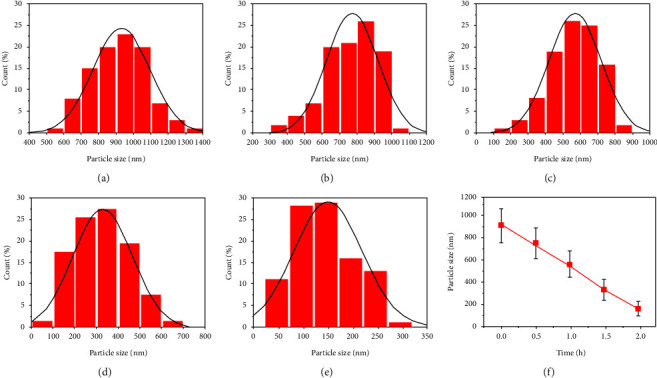
Particle size histograms of (a) Unmilled (0 h), (b) Milled @ 0.5 h, (c) Milled @ 1 h, (d) Milled @ 1.5 h, (e) Milled @ 2 h, (f) Evolution of the zinc oxide NPs' average size versus the milling time.

Figure 2 shows TEM images of ZNPs prepared via HSBM at various milling times. As shown, the milling process results in semispherical ZNPs with some local agglomeration. Furthermore, the average size of the nanoparticle distribution histograms (Figures 3(b), 3(c), 3(d), and 3(f)) is obtained by counting more than 100 particles using Image J software. Accordingly, the average particle sizes of the unmilled (0 h) and milled powders for 0.5 h, 1 h, 1.5 h, and 2 h are, respectively, 929 (±163), 767 (±145), 567 (±144), 327 (±98), and 148 (±68) nm (Figure 3(e)). Therefore, the HSBM technique successfully reduces particle size by about 85% after only 2 h of processing. The average NP size obtained by HSBM at 1000 rpm after 2 h is quite similar to that obtained by Salah et al. [25] (Table 1) by using the same technique at 300 rpm for 10 h. Consequently, HSBM reduces NPs by approximately 6 nm/min at 1000 rpm, compared to 25 nm/min at 300 rpm. Thus, this technique could produce results comparable to other sophisticated methods [28, 29]. Nevertheless, it is necessary to examine the effect of this process on the material structural properties.

#### 3.1.2. XRD Analysis

**Figure 4:**
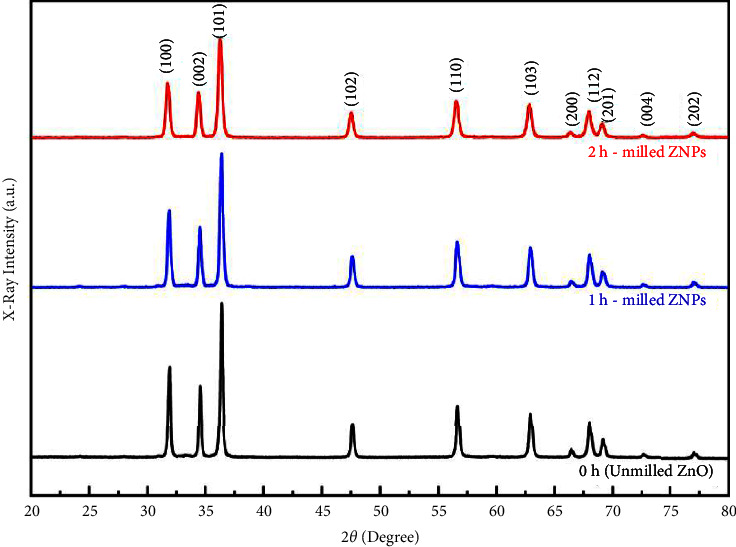
X-ray diffraction peaks of ZnO before and after the HSBM process.

Figure 4 shows the XRD patterns of selected samples before and after the milling process. They reveal hexagonal wurtzite structures. The prominent peaks located at around 32.8°, 34.58°, 36.08°, 47.58°, 56.88°, 63.08°, 66.58°, 68.08°, 69.08°, 72.58°, and 75.58° correspond to (100), (002), (101), (102), (110), (103), (200), (112), (201), (004), and (202) planes, respectively. As compared to the as-prepared ZnO sample, milled ZnO powder displayed the same diffraction pattern. Therefore, the milling process did not produce any phase transformations. As can be seen from Figure 4, the diffracted peaks become broader and less intense as the milling time increases. This might be due to the reduction in particle size, as suggested by Aruna and Rajam [46].

#### 3.1.3. FT-IR Spectroscopy

**Figure 5:**
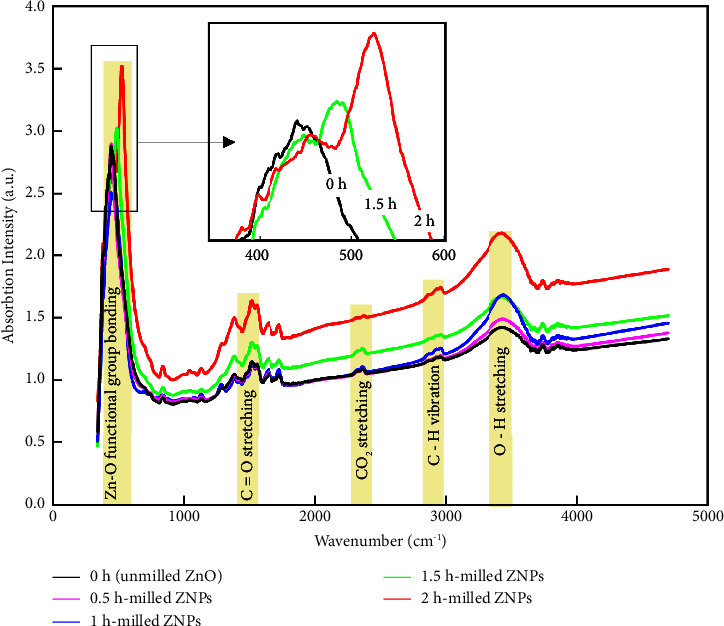
The FT-IR spectra of ZnO milled at different ball-milling time.

The FT-IR analysis of the various studied samples is shown in Figure 5. Significant absorption peaks can be observed at 1515, 2362, 2929, and 3420 cm^−1^. The IR bands at 3420 cm^−1^ are caused by stronger bounded adsorbed water molecules resulting from stretching OH–group vibrations. The band at 2929 cm^−1^ corresponds to the symmetric valency bands C–H, while the peak at around 2362 cm^−1^ identifies the O=C=O bond. It should be noted that as the particle size decreases, the absorbance increases. This is most likely due to the large surface area and volume ratio of nanocrystals in milled ZNPs. Indeed, in nanoparticles, the atomic arrangements on the borders differ greatly from those of the unmilled ZnO crystals, showing a certain magnitude of the disorder [47, 48]. In the abovementioned peaks, there is no noticeable change in band position (band shift); however, in the 300–600 cm^−1^ frequency band, a noticeable shift and distinct peak (450 cm^−1^) are evident. In this band, absorbance peaks are associated with stretching bonds between Zn and O, which correspond to the hexagonal phase of ZnO [49]. As shown in the zoomed section, HSBM resulted in a consistently shifting peak position with decreasing particle size. These discrepancies in vibrational modes have been associated with changes in the surface polarization charge created by changes in material size, as reported by Mo et al. [50] and Abdulkhadar and George [51]. The small grains have a high surface area ratio, allowing incident and scattered light to be absorbed efficiently. FT-IR fundamental absorption and vibration frequencies of atoms or groups of atoms in ZnO-milled materials are, therefore, size-dependent.

#### 3.1.4. Raman Spectroscopy

**Figure 6:**
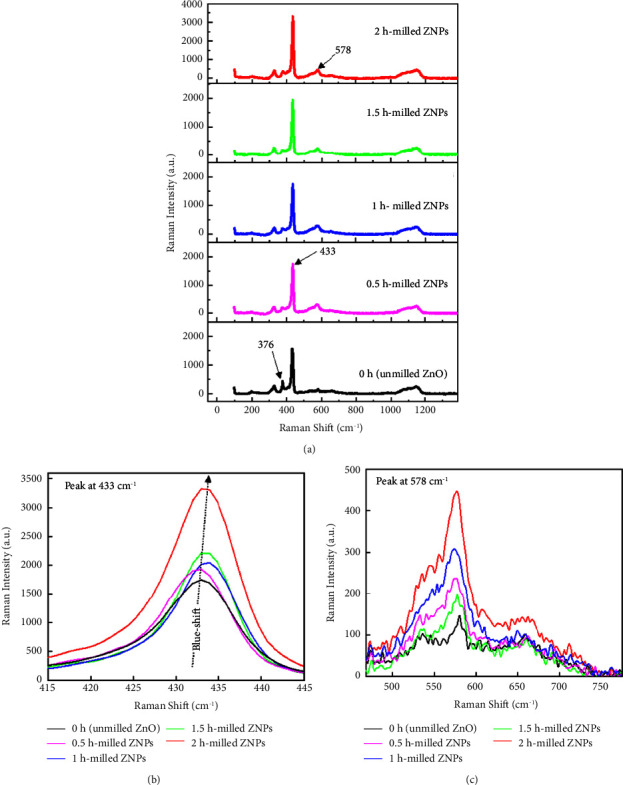
(a) Raman scattering spectra in the frequency domain 100–3500 cm^−1^ of milled ZNPs during 0.5, 1, 1.5, and 2 h. Note that the spectrum at 0 h corresponds to the unmilled ZnO powder. Zoom of 415–445 cm^−1^ (b) and 450–750 cm^−1^ (c) frequency domains.

ZNPs were also physically characterized using Raman spectroscopy (Figure 6(a)). Significant changes in peak position, intensity, or FWHM occur as a result of ball milling. A Raman peak at 376 cm^−1^ caused by bound excitonic transitions in ZnO reveals the strength of the polar lattice bonds associated with mode A_1_. According to Giri et al. [52], this peak disappears after 15 h of milling. The most prominent peak in the ZnO material occurred at 433 cm^−1^ (Figure 6(b)). This characteristic corresponds to the E_2_ high mode of the wurtzite phase. Another mode could be observed at 578 cm^−1^ (Figure 6(c)). According to Serrano et al. [53], this mode is caused by either oxygen vacancy, zinc interstitial, or their complexes. After milling, there is a blue shift of about 1 cm^−1^ for all main peaks compared with the ZnO starting powder. The blue shift indicates an increase in phonon frequencies interacting with incident photons, and the amount of shift determines the energy of the phonons in the material. The milling process would cause atoms in a crystal to move closer together or shorten their chemical bond lengths, compared with their normal positions and lengths in unstressed ZnO powder. Raman peak positions are, therefore, shifted to higher frequencies due to the possible compressive strains that arise from mechanical milling [54]. The Raman intensity depends on the particle size in the sample: it increases as the size of the ZNPs decreases due to diffuse reflection-induced optical losses in the case of larger particles. Similar behavior was observed by Wang et al. [54], and Hu et al. [55]. In their conclusion, the major factor is the improved diffuse reflectance: the smaller the particles and the wider the mismatch between the refractive indices of the particles and the medium, the greater the diffuse reflectance and the better the coupling between excitation and collection, resulting in an overall Raman signal increase [6].

#### 3.1.5. Zeta Sizer Analysis

**Figure 7:**
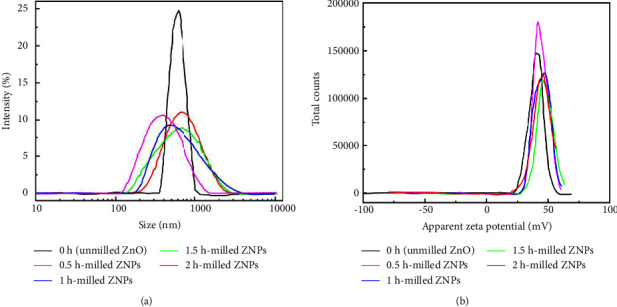
(a) ZnO size dependence, and (b) zeta potential dependence on milling time.

The Zetasizer instrument uses the dynamic light scattering (DLS) principle to identify the average size and the zeta potential of ZNPs dispersed in the base fluid. Comparatively to TEM, DLS can measure a larger number of particles (in millions). Therefore, it provides accurate data on particle sizes. The DLS allows the measurement of the hydrodynamic radius of the particles dispersed. In contrast, the TEM calculates the projected surface of the sample based on the number of electrons transmitted through it. In turn, the average particle size determined by DLS is typically much larger than that determined by TEM. As can be seen in Figure 7(a), the average size decreases as the milling time increases. Compared to other samples that are characterized by less intense and more broadened peaks, the unmilled sample (0 h) exhibits a narrow peak and scatters much light.

For a better understanding of the size distribution and homogeneity of ZNPs, the dimensionless Polydispersity Index (PDI) data are summarized in Table 2. The PDI is less than 0.4 in all samples, which indicates that ZNPs are in moderate dispersion in deionized water. It should be noted that aggregation will show an increase in the z-average size along with an increase in the PDI. Therefore, the z-average is highly sensitive to high-intensity scatters (big particles). These results confirm the previous results obtained from FT-IR and Raman analysis. To achieve the objective of our work and examine the stability of the ZNPs, zeta potential dependence was measured (Figure 7(b)). According to the guidelines for the classification of nanoparticle dispersion described in ref [53], ±0–10 mV, highly unstable; ±10–20 mV: relatively stable; ±20–30 mV: moderately stable; and ˃±30 mV: very stable, one can say ZNPs are highly stable.

### 3.2. Antimicrobial Activity

**Figure 8:**
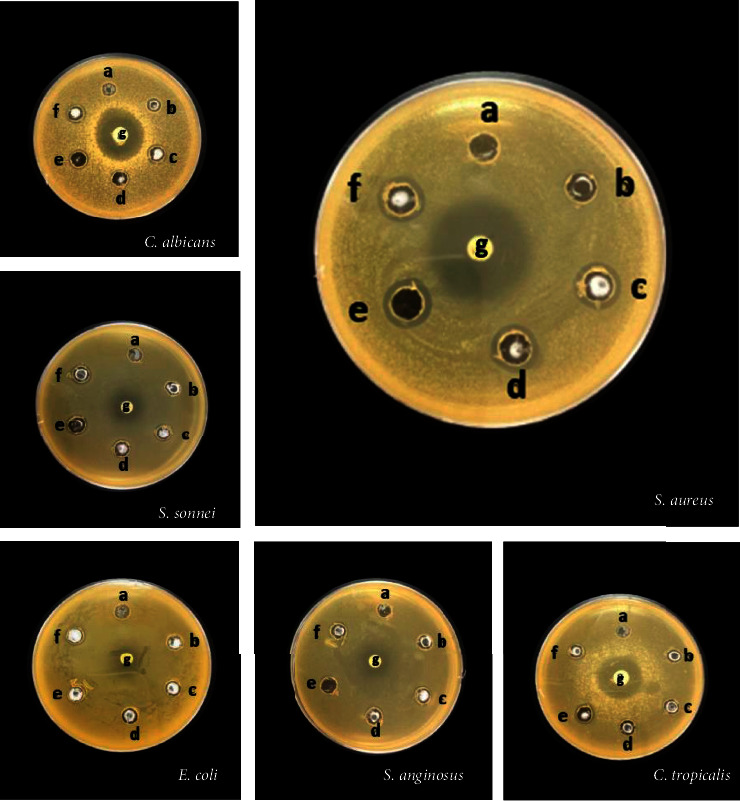
Antimicrobial activity of ZNPs at different milling times: b, 0 h; c, 0.5 h; d, 1 h; e, 1.5 h; and f, 2 h; and a, negative control; and g, positive control.

A test of antimicrobial activity revealed that ZNPs inhibited the growth of six out of eleven microorganisms. The results are shown in Figure 8 and Table 3. ZNPs milled under these conditions have broad-spectrum activity against Gram-negative/Gram-positive bacteria and candida. This inhibition is, however, dependent on both nanoparticle size and microorganism type. In Gram-negative bacteria, the bacterium *S. sonnei* was the most inhibited by the milled ZNPs. Followed by the *E. coli ATCC* 25922 which was inhibited only when the ZNPs were milled for 1.5 and 2 h, while *E. coli, K. pneumonia*, and *P. aeruginosa* were resistant to all milled and unmilled samples, in Gram-positive bacteria, both *S. aureus* and *S. anginosus* were inhibited only when ZNPs were milled for 0.5 h and 2 h. Moreover, *S. aureus* was the most susceptible, in which the inhibition zone size gradually increased from 11.8 ± 0.3 mm to 13.5 ± 0.5 mm in proportion to the increase in milling time from 0.5 h to 2 h, whereas the standard bacterium *S. aureus ATCC* 25923 was resistant to all milled and unmilled ZNPs. The tested candida showed similar results to the bacteria, which varied in their response to different milling times. The *C. albicans* was the most sensitive candida; it is inhibited by all the milled ZNPs. Followed by *C. tropicalis,* which was inhibited only by ZNPs ground for 1 h, 1.5 h, and 2 h, *C. knisei* was resistant to all nanoparticle sizes. ZnO bulk powder showed no significant antimicrobial activity.

### 3.3. Microorganism-ZNP Interaction Mechanisms

To interpret the results, we should look for nanoparticle-mediated toxicity through the interaction between nanoparticles and microorganisms. Indeed, results reported in Table 3 demonstrated that the antimicrobial activity of ZNPs is strongly dependent on the nanoparticle size, as it varies with the type of microorganism. Specifically, the smaller ZNPs exhibited the strongest antimicrobial activity and the largest inhibition zone. According to Miri et al. [15], the small size of NPs may allow a larger and better attachment to the microorganism cell wall due to the surface-to-volume ratio. Incontrovertibly, the antimicrobial activity of ZnO nanoparticles is a complex and dynamic process. Nevertheless, based on the experimental evidence and studies, ZNPs have a variety of mechanisms of action against microorganisms.

In their recent studies on the antifungal effects of synthesized ZNPs against *C. albicans*, Dananjaya et al. [14] and Miri et al. [15] observed damaged *C. albicans* cell membranes after treatment with ZNP size of 184 nm and 40–80 nm, respectively. The microorganism membrane smoothness was affected by ZnO nanoparticles, resulting in surface bulges taking on an unusual shape and compromising membrane integrity. They concluded that the antifungal activity of ZNPs arises from the nanoparticle penetration through the plasma membrane, which leads to metabolic disturbance, and ultimate cell death. In our study, *C. albicans* was the most sensitive to ZNPs ranging in size from about 767 to 148 nm. Therefore, we can conclude that the penetration of NPs of smaller size through the candida membrane pores is not the only attack mechanism. Especially when considering that the cell wall porosity is significantly lower in *C. albicans* compared to other candida [56], indeed, at this point, the current knowledge about the *C. albicans* cell wall and its interaction with external agents or cells cannot be extrapolated to *C. tropicalis* and *C. knisei* [56], because these species have different cell wall compositions that may be responsible for the obtained results.

For bacteria, the penetration procedure of Zn^2+^ depends on the bacterial group. Indeed, differences between Gram-positive and Gram-negative bacteria are primarily attributed to their cell walls: the Gram-negative cell wall is characterized by the presence of an outer membrane, a thinner peptidoglycan layer, and extensive periplasmic space surrounding the peptidoglycan. The uppermost layer of the outer membrane has lipopolysaccharide, while the innermost layer is a typical phospholipid. Gram-positive cells consist of a single thick layer of walls made up of peptidoglycan, teichoic acid, and lipoteichoic acid. They lack an outer membrane and porin and have a narrow periplasmic space. The membrane channels formed by porin proteins in the outer membranes of Gram-negative cells render them less penetrable than Gram-positive cells. Accordingly, the different susceptibility to zinc oxide nanoparticles in the two bacterial groups, as shown in Table 3, could be explained by differences in the bacterial cell walls. For instance, among the six Gram-negative bacteria tested, only *S. sonnei* showed the largest inhibition zone of about 11.3 ± 0.6 mm with ZnO nanoparticles measuring 148 nm in size (milled for 2 h) comparable with the results shown by erythromycin, used as a positive control. A similar inhibition in Gram-negative bacteria was previously obtained for ZNPs of average particle sizes ranging 600–30 nm [9], 90–200 nm [40], 80 nm [13], 12–212 nm [8], 130–50 nm [57], and 5–38 nm [7]. Nevertheless, since the synthesis process of ZNPs differs in these studies, slight differences in the inhibition activity should be mentioned.

Gram-positive bacteria were more susceptible to the antimicrobial action of ZnO: In detail, *S. aureus* and *S. anginosus* were influenced by all milled materials, while the inhibition zone increased as the particle size decreased. However, the largest inhibition zone of 13.2 ± 0.3 mm was measured against *S. aureus* bacteria (Figure 8). The size of the inhibition zone observed in the present work is similar to that measured by Bazant et al. [58] (inhibition zone, 11−14 mm) and less than that measured by Zubair and Akhtar [59] (inhibition zone, 30 mm) for ZNPs of average size 11–18 nm and Mostafa [60] (inhibition zone, 24.5 mm) for ZNPs of size <100 nm. When the bactericidal activity of ZnO NPs against *S. aureus* was evaluated by monitoring cell respiration [13] and microscopic observations [7], it was suggested that the mechanism involved in the toxicity of this bacteria is the result of damage to the cellular structure through holes formed in the cell wall with extravasation of the intracellular contents. Nevertheless, the toxicity may also be induced by zinc ions as a heavy metal's effects. Indeed, microorganisms are susceptible to oxidative stress due to ROSs generation produced by ZnO nanoparticles, which inhibits protein synthesis and DNA replication. In this reaction, ROSs could be the superoxide anion (O_2_^·−^), hydrogen peroxide (H_2_O_2_), and the hydroxyl radical (HO^•^), as the pathways of bactericidal action. From another perspective, Zn^2+^ ions released from the dissolution of ZnO in the aqueous solution can also damage the cytoplasmic membrane. Due to its specific affinity for the sulfur group, the Zn^2+^ ion inhibits glycolysis through thiol group oxidation [61]. Nevertheless, according to all the discussed results, particles' size is not the only determinant factor in ZNPs' antimicrobial or antifungal activity but also the shape and concentration of ZNPs.

## 4. Conclusions

In this study, ZnO nanoparticles were produced by milling HSBM at 1000 rpm for short periods. XRD, TEM, FT-IR, Raman spectroscopy, and Zetasizer techniques were used to determine the induced structural and spectral modifications of ZNPs during the HSBM process. TEM results showed a gradual decrease of 6 nm/min in particle size with increased milling time. As particle size increases, X-ray diffracted patterns become less intense and broader, confirming the absence of phase transformation. FT-IR spectra show a clear discrepancy when particle size is reduced, resulting from dipolar interactions, interfacial effects, surface, amorphousness, and surface-free energy. The size of particles also affects Raman responses. Indeed, the broadening of the principal peak that occurred at 433 cm^−1^ confirms a change in the band structure of ZNPs after milling. The Zeta Sizer measurements showed that ZNPs are highly stable and dispersed in water (PDI < 0.4) when using a CTAB surfactant element. This suggests that the HSBM technique is quite suitable for large-scale production of this type of nanoparticle. The results showed that the synthesized nanoparticles possessed a broad spectrum of activity against several human pathogenic microorganisms, mainly *S. sonnei in* Gram-negative bacteria. *S. aureus* was the most susceptible Gram-positive bacteria, and *C. albicans* was the most susceptible candida.

## Figures and Tables

**Table 1:** Synthesis methods of ZNPs and tested microorganisms.

Year	Reference	Technique	ZNPs size	Tested microorganisms
2022	El-Shaer et al. [18]	Arc discharge	20 nm	(i) *Escherichia coli*
				(ii) *Klebsiella pneumoniae*
				(iii) *Proteus mirabilis*
				(iv) *Staphylococcus aureus*

2020	Seray et al. [30]	Solvent casting	<100 nm	(i) *Staphylococcus aureus*
				(ii) *Pseudomonas aeruginosa*
				(iii) *Bacillus subtilis*

2020	Zanet et al. [31]	Combustion method/chemical vapor synthesis	50–100 nm	(i) *Listeria monocytogenes*
				(ii) *Bacillus subtilis*
				(iii) *B. subtilis*
				(iv) *Staphylococcus aureus*
				(v) *Escherichia coli*
				(vi) *Salmonella enterica*
				(vii) *Escherichia coli*
				(viii) *Saccharomyces cerevisiae*

2019	Yusof et al. [32]	Microwave heating (using chitosan as a stabilizing agent)	50–130 nm	(i) *Staphylococcus aureus*
				(ii) *Escherichia coli*

2019	Dadi et al. [33]	Sol-gel method (using acetate as a precursor)	3 nm	(i) *Staphylococcus aureus*
				(ii) *Escherichia coli*
				(iii) *Pseudomonas aeruginosa*

2019	Baek et al. [34]	Encapsulation in an alginate biopolymer solution	120–36 nm	(i) *Escherichia coli*
				(ii) *Pseudomonas aeruginosa*

2019	Akbar et al. [35]	Chemical synthesizing (using ethanolic colloidal solution)	20 nm	(i) *Salmonella typhimurium*
				(ii) *Staphylococcus aureus*

2018	Zang et al. [36]	Biological degradation (in light/dark medium mill with a pH value of 7.2)	30 nm	(i) *Escherichia coli*

2016	Zohourvahid Karimi and Ansari [37]	Solution method (SM)/milling method (MM) (planetary ball milling: rate of 280 rpm for 20 min)	9–11 nm (SM)	(i) *Staphylococcus aureus*
			26–29 nm (MM)	(ii) *Escherichia coli*

2016	Manzoor et al. [38]	Mechano-chemically @ 250°C	<20 nm	(i) *Enteropathogenic*
				(ii) *Escherichia coli*
				(iii) *Campylobacter jejuni*
				(iv) *Vibrio cholerae*
				(v) *Staphylococcus aureus (MRSA)*

2016	Isaei et al. [39]	Sol-gel method (in low temperature)	30 nm	(i) *Pseudomonas aeruginosa*

2015	Jiang et al. [40]	Coating of PVC-based films by ZNPs	100–1000 nm	(i) *Staphylococcus aureus*
				(ii) *Escherichia coli*
				(iii) *Aspergillus flavus*
				(iv) *Penicillium citrinum*

2012	Azam et al. [41]	Chemical heating @ 400°C	26 nm	(i) *Escherichia coli*
				(ii) *Staphylococcus aureus*
				(iii) *Pseudomonas aeruginosa*
				(iv) *Bacillus subtilis*

2011	Espitia et al. [42]	Mechanochemical processing/physical vapor synthesis	40–24 nm	(i) *Escherichia coli*
				(ii) *Staphylococcus aureus*

2011	Bhadra et al. [43]	Coprecipitation method	80 nm	(i) *Escherichia coli*

2011	Premanathan et al. [44]	Wet chemical method	20–30 nm	(i) *Escherichia coli*
				(ii) *Pseudomonas aeruginosa*
				(iii) *Staphylococcus aureus*

2011	Salah et al. [25]	Ball milling	600–30 nm	(i) *No* tested microorganisms

2010	Li et al. [45]	Coating of PVC-based films by ZNPs	50–500 nm	(i) *Escherichia coli*
				(ii) *Staphylococcus aureus*

2010	Jalal et al. [10]	Microwave decomposition	37–47 nm	(i) *Escherichia coli*

**Table 2:** PDI of different studied samples.

Milling time (h)	0	0.5	1	1.5	2
PDI	0.25	0.20	0.22	0.25	0.19

**Table 3:** Antimicrobial activity of ZNPs against some pathogenic bacteria and yeasts using well diffusion technique.

Test microorganisms	Inhibition zone (mm) using well diffusion technique ± SD					Positive control		Negative control
	0.0 h ZNPs	0.5 h ZNPs	1.0 h ZNPs	1.5 h ZNPs	2 h ZNPs	E 15 *μ*	Nystatin	
*Gram-negative bacteria*								
*E. coli* ATCC25922	0	0	0	10 ± 0.5	10.5 ± 0.5	12.5 ± 0.5	—	0
*E. coli*	0	0	0	0	0	7.7 ± 0.6	—	0
*K. pneumonia*	0	0	0	0	0	7.7 ± 0.6	—	0
*P. aeruginosa*	0	0	0	0	0	8.3 ± 0.6	—	0
*S. sonnei*	0	9.5 ± 0.5	10 ± 0.5	11.3 ± 0.3	11.3 ± 0.6	12.5 ± 0.5	—	0

*Gram-positive bacteria*								
*S. aureus* ATCC 25923	0	0	0	0	0	0	—	0
*S. aureus*	0	11.8 ± 0.3	12.6 ± 0.6	13.5 ± 0.5	13.2 ± 0.3	25.8 ± 0.8	—	0
*S. anginosus*	0	10.2 ± 0.3	10.3 ± 0.6	11.8 ± 0.3	11.5 ± 0.5	20 ± 0.8	—	0

*Candida*								
*Can. knisei*	0	0	0	0	0	—	0	0
*Can. tropicalis*	0	0	9.8 ± 0.3	11.8 ± 0.3	11.7 ± 0.3	—	22.8 ± 0.8	0
*Can. albicans*	0	10.2 ± 0.3	10.2 ± 0.3	11.7 ± 0.3	11.5 ± 0.5	—	26.5 ± 0.5	0
Total antimicrobial activity	0	4	5	6	6	—	—	—

## Data Availability

Data are available on request from the corresponding author.
